# The maternal antibody against diphtheria, tetanus and pertussis showed distinct regional difference in China

**DOI:** 10.1186/s12887-019-1860-5

**Published:** 2019-12-07

**Authors:** Qinghong Meng, Qinghui Qian, Li Li, Dandan Liu, Wei Gao, Lin Yuan, Kaihu Yao

**Affiliations:** 1 Key Laboratory of Major Diseases in Children, Ministry of Education, National Key Discipline of Pediatrics (Capital Medical University), Beijing Pediatric Research Institute, Beijing Children’s Hospital, Capital Medical University, National Center for Children’s Health, Beijing, 100045 China; 2 Department of Pediatrics, Qianjiang Central Hospital, Chongqing, 409000 China; 3 Department of Neonatology, Shunyi Women and Children’s Hospital of Beijing Children’s Hospital, Beijing, 101320 China

**Keywords:** Passive transferred antibodies, Neonate, DTP, Shunyi, Qianjiang

## Abstract

**Background:**

Passive transferred antibodies to the fetus play an essential role on protecting neonates and young infants until infant vaccination is more efficacious. However, very little is known about the discrepancy of DTP vaccine associated antibodies level in neonates from different economic areas in China.

**Methods:**

In 2018, 200 neonates hospitalized in Shunyi Women and Children’s Hospital in Beijing, and 238 neonates hospitalized in Qianjiang Central Hospital located in the southwestern mountainous areas were included in this study. Antibodies specific for the antigens covered by DTP vaccine were determined using ELISA Kits (Euroimmun, Lübeck, Germany). The cut off value of ≥0.1 IU/ml (anti-diphtheria, anti-Dtx), > 0.1 IU/ml (anti-tetanus, anti-Ttx) and > 40 IU/ml (anti-pertussis toxin, anti-Ptx) were used to assess the percentage of protected neonates, respectively.

**Results:**

The antibody levels in the neonates from Qianjiang (0.04 IU/ml for anti-Dtx IgG and 0.07 IU/ml for anti-Ttx IgG) were significantly lower than those from Shunyi (0.12 IU/ml for anti-Dtx IgG and 0.18 IU/ml for anti-Ttx IgG). The prevalence of protective anti-Dtx and anti-Ttx IgG were lower in the neonates from Qianjiang (7.1% for anti-Dtx IgG and 7.6% for anti-Ttx IgG) than in those from Shunyi (30.5% for anti-Dtx and 38.5% for anti-Ttx). The neonates from Qianjiang also had lower detectable rate of anti-Dtx (57.5%) and anti-Ttx IgG (55.8%) than neonates from Shunyi (97.5% for anti-Dtx and 71.0% for anti-Ttx). However, the detectable rate of anti-Ptx IgG in neonates from Qianjiang (39.9%) was higher significantly than in those from Shunyi (30.5%). Two neonates from Qianjiang have anti-PT IgG ≥100.0 IU/ml, which suggested that their mothers have a recent pertussis course.

**Conclusions:**

The regional discrepancy of the protective antibody rates might be caused by different vaccine coverage and pertussis exposure, which suggested the importance of Tdap booster immunization for pregnant women or women at childbearing age, those living undeveloped areas in particular.

## Background

Immunization is the most successful and cost-effective interventions for prevention of many infectious diseases. It has recorded remarkable successes in eradication of polio, smallpox, measles and rubella from certain regions of the world, and substantial reductions in diphtheria, tetanus and pertussis-related morbidity and mortality [1].

Diphtheria, tetanus and pertussis are vaccine-preventable respiratory infectious diseases caused by *Corynebacterium diphtheria1*, *Clostridium tetani* and *Bordetella pertussis*, respectively [2–4]. Diphtheria and tetanus immunization has resulted in a significantly decrease in the incidence of these diseases. No case of diphtheria has been reported in China for nearly 20 years. The incidence of neonatal tetanus in China has also dramatically decreased from 1585 cases per 100,000 population in 2000 to 1 cases per 100,000 population in 2017. However, a resurgence of pertussis occurred in China. According to reports from National Health Commission of the People’s Republic China, a total of 22,466 cases were reported in 2018. From January to October in 2019, the number of reported pertussis cases had already reached to 27,660. According to our previous research, from May 2013 to September 2014, among the 99 culture-confirmed pertussis patients in Beijing Children’s Hospital, 42 cases (42.4%) were less than 3 months of age [5]. The hospitalization rate and deaths are particularly high in young infants. Neonatal immunization is usually unsuccessful because of the immature immune system [6]. Therefore, passive transferred maternal antibodies play an important role on reducing the risk of infection until infant vaccination is more efficacious [7].

In China, a routine national immunization program was implemented in 1978. The standard diphtheria-tetanus-whole cell pertussis (DTwP) immunization schedule included three doses of primary DTwP vaccination at 3rd, 4th and 5th months of age and a booster dose at 18–24 months. Since implementation of the Expanded Program on Immunization (EPI) of China in 2007, a combined diphtheria-tetanus-acellular pertussis (DTaP) vaccine was introduced, both DTwP and DTaP were used [8, 9]. Another dose of DT vaccine had been supplemented at 6 years old. In order to reduce the incidence of pertussis in infants and young children, Tdap booster during pregnancy is widely implemented in many developed countries [10, 11]. However, our vaccination program has remained unchanged since 1980s, and no maternal Tdap immunization was introduced into China. Therefore, the maternal antibody associated with DTP vaccine mainly depends on childhood immunizations and nature infections in China. According to our previous research, the prevalence of protective anti-diphtheria (Dtx) and anti-tetanus (Ttx) IgG in cord sera were positively associated with pregnant women’ birth places [12]. Anti-Ttx were used as an indicator of vaccination with tetanus toxoid-containing vaccine since natural immunity following tetanus disease does not occur as the quantity of tetanus toxin required to produce disease is inadequate to induce an immune response [1]. It reminded us that the anti-Dtx and anti-Ttx IgG levels was acquire through DTP vaccination, and the difference in subgroup was attribute to the inequalities of immunization coverage. The DTP coverage of the primary three doses has been increasing in the whole country since 2009. However, at present, the women of child-bearing age in China were mainly born in 1970s–1990s, the real DTP coverage have relationships with local economic, medical and education level.

Beijing is the capital of the People’s Republic of China, with 21 million permanent residents. Shunyi District is one of urban area of Beijing. Chongqing is a municipality in southwest of China, with 30 million permanent residents. Qianjiang is one of the districts of Chongqing, which were located in the southwestern mountainous area. The aim of this study was to determine DTP vaccine associated antibodies in neonates from two geographically distant areas.

## Methods

### Serum samples

In 2018, 200 neonates hospitalized in the neonatal ward of Shunyi Women and Children’s Hospital of Beijing Children’s Hospital, and 238 neonates hospitalized in the department of pediatrics of Qianjiang Central Hospital were included in this study. They were hospitalized for noninfectious disease, and those neonates with any suspicious infection were not involved in the study. A routinely peripheral blood sample was obtained at admission. All serum samples were frozen at − 20 °C until analysis.

### Serological testing

Anti-Dtx, anti-Ttx and anti-pertussis toxin (Ptx) IgG levels were measured by enzyme-linked immunosorbent assay using commercial ELISA kits (Euroimmun, Lübeck, Germany), and antibody activity was expressed in international units per milli-liter (IU/ml).

### Cut-off values

Results were interpreted based on the manufacturer’s protocol and previous studies [13–15]. Anti-Dtx level < 0.1 IU/ml was defined as no protection, the level of 0.1–1.0 IU/ml was defined as short term protection, a value > 1.0 was defined as long term protection (The values of > 1.0–1.5 IU/ml, > 1.5–2.0 IU/ml, and > 2.0 IU/ml mean booster after 5 years, 7 years and 10 years). Anti-Ttx level ≤ 0.1 IU/ml was defined as no protection, the level of > 0.1–0.5 IU/ml was defined as short term protection, a value > 0.5 was defined as long term protection (The values of > 0.5–1.0 IU/ml, > 1.0–5.0 IU/ml, and > 5.0 IU/ml mean booster after 3 years, 5 years and 8 years). The anti-Ptx level < 40.0 IU/ml was defined as negative, a value ≥100.0 IU/ml indicates an acute infection or recent vaccination (positive), a value between 40.0 and 100.0 was equivocal.

### Data analysis

The distribution of serum antibodies in different age groups were expressed as mean concentration with 95% confidence interval (CI). For statistical analysis, antibody concentrations below the lower limit for quantitation were assigned as half the lower limit of quantitation (0.005 IU/ml for anti-Dtx and anti-Ttx, and 2.5 IU/ml for anti-Ptx). The value of anti-Ttx level ≥ 5.0 IU/ml and anti-PT level ≥ 200.0 IU/ml was counted as 5.0 IU/ml and 200.0 IU/ml, respectively. The seroprevalence among different groups was compared with the chi-square test. The difference in antibody concentration among different age groups was tested by the Wilcoxon/Krusakal Wallis test. Data was analyzed using the JMP (version 10.0) and SPSS (version 17.0). *P* ≤ 0.05 was considered statistically significant.

## Results

### Demographic characteristics

In total, 438 neonates (1–28 day) were enrolled in the study. Among them, 200 neonates were collected in Shunyi Women and Children’s Hospital of Beijing Children’s Hospital (95 females, 105 males; 154 term infants, 46 preterm infants), and another 238 neonates were collected in Qianjiang Central Hospital of Chongqing (107 females, 131 males; 225 term infants, 13 preterm infants). A total of 114 neonates from Shunyi Women and Children’s Hospital of Beijing Children’s Hospital, and 153 neonates from Qianjiang Central Hospital of Chongqing were born naturally, respectively.

### Seroprevalence of anti-Dtx IgG

The mean concentration of anti-Dtx IgG was 0.08 IU/ml (95% CIs: 0.06–0.09). Higher mean concentration were found in Shunyi group (*P* < 0.0001) (Table 1). In both Shunyi and Qianjiang groups, the mean concentration did not differ significantly between males and females, between term infants and preterm infants, or between natural childbirth and C-section (Tables 2 and 3). The distributions of anti-Dtx IgG were presented in Table 4. Compared with neonates from Shunyi (2.5%), the undetectable rate of anti-Dtx IgG (< 0.01 IU/ml) in neonates from Qianjiang (42.9%) was higher significantly (χ^2^=95.876, *P* < 0.0001). While, the rate of neonates with basic protection (0.1–1.0 IU/ml) from Qianjiang were also lower significantly (χ^2^ = 41.016, *P* < 0.0001). Only 1 neonate from Shunyi showed long term protection (> 1.0 IU/ml), and no neonate from Qianjiang showed long term protection (Table 4).

**Table 1:** The mean concentration and 95% CI of anti-Dtx, anti-Ttx and anti-Ptx IgG in neonates hospitalized in the neonatal ward

Group	N	Mean (95%CI) (IU/ml)		
		Anti-Dtx	Anti-Ttx	Anti-Ptx
Shunyi	200	0.12 (0.09–0.15)	0.18 (0.12–0.25)	6.31 (5.24–7.38)
Qianjiang	238	0.04 (0.02–0.06)	0.07 (0.02–0.11)	7.75 (5.74–9.76)
*P*		< 0.0001	< 0.0001	0.1134
Total	438	0.08 (0.06–0.09)	0.12 (0.08–0.16)	7.09 (5.90–8.29)

**Table 2:** The demographic characteristics of neonates from Shunyi and mean concentration of anti-Dtx, anti-Ttx and anti-Ptx IgG

Group	N	Mean (95%CI) (IU/ml)		
		Anti-Dtx	Anti-Ttx	Anti-Ptx
Sex				
Female	95	0.11 (0.08–0.14)	0.14 (0.08–0.20)	5.47 (4.14–6.79)
Male	105	0.13 (0.08–0.18)	0.22 (0.11–0.33)	7.08(5.42–8.74)
*P*		0.4907	0.2415	0.1386
Birth				
Term infant	154	0.13 (0.10–0.17)	0.21(0.12–0.29)	6.22 (5.01–7.44)
Premature infant	46	0.08 (0.05–0.11)	0.12 (0.07–0.17)	6.62 (4.27–8.96)
*P*		0.1106	0.2962	0.7608
Delivery mode				
Spontaneous labor	114	0.12 (0.09–0.15)	0.20 (0.10–0.31)	5.92 (4.65–7.18)
Cesarean delivery	86	0.12 (0.07–0.17)	0.15 (0.10–0.21)	6.84 (4.98–8.71)
*P*		0.8609	0.4333	0.3770
Total	200	0.12 (0.09–0.15)	0.18 (0.12–0.25)	6.31 (5.24–7.38)

**Table 3:** The demographic characteristics of neonates from Qianjiang and mean concentration of anti-Dtx, anti-Ttx and anti-Ptx IgG

Group	N	Mean (95%CI) (IU/ml)		
		Anti-Dtx	Anti-Ttx	Anti-Ptx
Sex				
Female	107	0.03 (0.02–0.05)	0.04(0.02–0.07)	9.25(5.05–13.46)
Male	131	0.05 (0.02–0.08)	0.08(0.01–0.16)	6.52 (5.23–7.82)
*P*		0.4938	0.3906	0.1841
Birth				
Term infant	225	0.04 (0.02–0.06)	0.07 (0.02–0.11)	7.83 (5.71–9.95)
Premature infant	13	0.03 (0.01–0.05)	0.05 (0.01–0.09)	6.32 (2.52–10.11)
*P*		0.7483	0.8564	0.7365
Delivery mode				
Spontaneous labor	153	0.04 (0.02–0.07)	0.07 (0.01–0.13)	7.34 (5.65–9.03)
Cesarean delivery	85	0.04 (0.02–0.06)	0.07 (0.03–0.10)	8.48 (3.68–13.29)
*P*		0.8817	0.9860	0.5937
Total	238	0.04 (0.02–0.06)	0.07 (0.02–0.11)	7.75 (5.74–9.76)

**Table 4:** The distribution of anti-Dtx, anti-Ttx and anti-Ptx IgG in neonates hospitalized in the neonatal ward

Antibody level	N, Prevalence (95% CI)				χ^2^, *P*
	Shunyi		Qianjiang		
Anti-Dtx IgG					
< 0.01 IU/ml	5	2.5% (1.1–5.7%)	102	42.9% (36.7–49.2%)	χ^2^ = 95.876, *P* < 0.0001
0.01- < 0.1 IU/ml	134	67.0% (60.2–73.1%)	119	50.0% (43.7–56.3%)	χ^2^ = 12.873, *P* = 0.0003
0.1–1.0 IU/ml	60	30.0% (24.1–36.7%)	16	6.72% (4.2–10.6%)	χ^2^ = 41.016, *P* < 0.0001
> 1.0–1.5 IU/ml	0	0 (0–1.9%)	0	0 (0–1.6%)	–
> 1.5–2.0 IU/ml	0	0 (0–1.9%)	1	0.4% (0.1–2.3%)	χ^2^ = 0.842, *P* = 0.3588
> 2.0 IU/ml	1	0.5% (0.1–2.8%)	0	0 (0–1.6%)	χ^2^ = 1.193, *P* = 0.2748
Anti-Ttx IgG					
< 0.01 IU/ml	58	29.0% (23.2–35.6%)	105	44.2% (38.0–50.5%)	χ^2^ = 10.63, *P* = 0.0011
0.01–0.1 IU/ml	85	42.5% (35.9–49.4%)	115	48.3% (42.1–54.6%)	χ^2^ = 1.483, *P* = 0.2233
> 0.1–0.5 IU/ml	36	18.0% (13.3–23.9%)	17	7.1% (4.5–11.1%)	χ^2^ = 12.044, *P* = 0.0005
> 0.5–1.0 IU/ml	16	8.0% (5.0–12.6%)	1	0.4% (0.1–2.3%)	χ^2^ = 16.737, *P* < 0.0001
> 1.0–5.0 IU/ml	4	2.0% (0.8–5.0%)	0	0 (0–1.6%)	χ^2^ = 4.804, *P* = 0.0284
> 5.0 IU/ml	1	0.5% (0.1–2.8%)	0	0 (0–1.6%)	χ^2^ = 1.193, *P* = 0.2748
Anti-Ptx IgG					
< 5.0 IU/ml	139	69.5% (62.8–75.5%)	143	60.1% (53.8–66.1%)	χ^2^ = 4.202, *P* = 0.0404
5.0- < 40.0 IU/ml	59	29.5% (23.6–36.2%)	90	37.8% (31.9–44.1%)	χ^2^ = 3.348, *P* = 0.0673
40.0- < 100.0 IU/ml	2	1.0% (0.3–3.6%)	3	1.3% (0.4–3.6%)	χ^2^ = 0.065, *P* = 0.7982
≥ 100.0 IU/ml	0	0 (0–1.9%)	2	0.8% (0.2–3.0%)	χ^2^ = 1.688, *P* = 0.1932

### Seroprevalence of anti-Ttx IgG

The mean concentration of anti-Dtx IgG was 0.12 IU/ml (95% CIs: 0.08–0.16). Higher mean concentration were found in Shunyi group (*P* < 0.0001) (Table 1). In both Shunyi and Qianjiang groups, the mean concentration did not differ significantly between males and females, between term infants and preterm infants, or between natural childbirth and C-section (Tables 2 and 3). The distributions of anti-Ttx IgG were presented in Table 4. Compared with neonates from Shunyi (29.0%), the undetectable rate of anti-Ttx IgG (< 0.01 IU/ml) in neonates from Qianjiang (44.2%) was higher significantly (χ^2^ = 10.63, *P* = 0.0011). While, the rate of neonates with basic protection (> 0.1–0.5 IU/ml) from Qianjiang were lower significantly (χ^2^ = 12.044, *P* = 0.0005). The proportions with long term protection (> 1.0–5.0 IU/ml) from Qianjiang were also lower significantly (χ^2^ = 4.804, *P* = 0.0284). No neonate from Qianjiang showed long term protection (Table 4).

### Seroprevalence of anti-Ptx IgG

The mean concentration of anti-Ptx IgG was 7.09 IU/ml (95% CIs: 5.90–8.29). There was no significant difference in mean concentration between Shunyi and Qianjiang groups (*P* = 0.2395) (Table 1). In both Shunyi and Qianjiang groups, the mean concentration did not differ significantly between males and females, or between term infants and preterm infants (Tables 2 and 3). The distributions of anti-Ptx IgG were presented in Table 4. A total of 69.5% neonates from Shunyi, and 60.1% of neonates from Qianjiang had anti-Ptx IgG that was below the lower limit of detection (< 5.0 IU/ml), respectively. Compared with neonates from Shunyi, the undetectable rate of anti-Ptx IgG (< 5.0 IU/ml) in neonates from Qianjiang was lower significantly (χ^2^=4.202, *P*=0.0404). There were no significant differences in the proportions of neonates with an equivocal (χ^2^ = 0.065, *P* = 0.7982) and seropositive (χ^2^ = 1.688, *P* = 0.1932) level between these two regions.

### Protective antibody levels in sera of neonates

Neonates with protective antibody were presented in Table 5. A total of 30.5% (24.5–21.7%), 38.5% (22.7–35.1%) and 1.0% (0.3–3.6%) of neonates from Shunyi had protective concentrations of anti-Dtx IgG, anti-Ttx IgG, and anti-Ptx IgG, respectively. A total of 7.1% (4.5–11.1%), 7.6% (4.8–11.6%) and 2.1% (0.9–4.8%) of neonates from Qianjiang had protective concentrations of anti-Dtx IgG, anti-Ttx IgG, and anti-Ptx IgG, respectively. Compared with neonates from Shunyi, the protective rate of anti-Dtx (χ^2^ = 21.788, *P* < 0.0001) and anti-Ttx (χ^2^ = 33.569, *P* < 0.0001) in neonates from Qianjiang was lower significantly. However, no significant differences between the protective rate of anti-Ptx were observed between Shunyi and Qianjiang groups (χ^2^=0.837, *P* <0.3601) (Table 5).

**Table 5:** Prevalence of protective DTP associated antibody in neonates hospitalized in the neonatal ward

Group	N	Protective % (95% CI)		
		Anti-Dtx (≥0.1 IU/ml)	Anti-Ttx (> 0.1 IU/ml)	Anti-Ptx (> 40 IU/ml)
Shunyi	200	30.5% (24.5–37.2%)	38.5% (22.7–35.1%)	1.0% (0.3–3.6%)
Qianjiang	238	7.1% (4.5–11.1%)	7.6% (4.8–11.6%)	2.1% (0.9–4.8%)
χ^2^, *P*		χ^2^ = 21.788, *P* < 0.0001	χ^2^ = 33.569, *P* < 0.0001	χ^2^ = 0.837, *P* < 0.3601
Total	438	17.8% (14.5–21.7%)	17.1% (13.9–20.9%)	1.6% (0.8–3.2%)

## Discussion

The different pattern of DTP-associated antibodies in two geographically distant primary hospitals was observed: lower detectable rate and protective rate of anti-Dtx and anti-Ttx IgG in Qianjiang; but higher detectable rate of anti-Ptx IgG. Although there were protective antibody surveys in different regional studies, few study focused on the possible regional discrepancy.

There is variety in defining protective concentrations for anti-Dtx and anti-Ttx IgG in the international literature. Both 0.01 IU/ml [16–18] and 0.1 IU/ml [15, 19] were used in various studies. For safety reasons, the higher cut off value (0. 1 IU/ml) was adopted in our study. This criterion is also in line with the manufacturer’s instructions. The percentages of neonates from Shunyi with protective anti-Dtx (30.5%) and anti-Ttx (38.5%) IgG level were consistent with our previous studies among cord sera carried in Shunyi (a total of 38.7 and 26.8% of neonates had protection for diphtheria and tetanus) [12]. The prevalence of protective anti-Dtx and anti-Ttx IgG were lower in the neonates from Qianjiang (7.1% for anti-Dtx IgG and 7.6% for anti-Ttx IgG) than in those from Shunyi. There was no diphtheria case reported in the recent years in China, therefore, the nature boosting by circulating *Corynebacterium diphtheria1* less occurred. While, natural immunity following *Clostridium tetani* infection does not occur. We speculated that this discrepancy could be caused by greater proportion of DTP vaccination in mothers from Shunyi. As a big country, there were some differences on immunization approaches, immunization coverage and control of infectious diseases in geographically distant areas. As the capital of the People’s Republic of China, Beijing had better immunization coverage and control of infectious diseases. Therefore, women of child-bearing age and their neonates had better protection for infectious diseases. The immunization coverage of DTP vaccination has been increasing, being greater than 90% since 1990, however, the vaccination coverage was low before the 1980s and only 58% in 1983 [20]. At present, the women of child-bearing age in China were mainly born in 1970s–1990s. Therefore, women of child-bearing age who were born in 1970s–1980s were still generally lack protection for diphtheria and tetanus, even in Beijing. This situation will be more serious in remote regions or lower income regions.

In our study, nearly all neonates had no protection against pertussis. No significant differences between the rates of unprotected neonates were observed between Shunyi (99.0%) and Qianjiang groups (97.9%). It was similar with our previous investigation in cord blood samples, which revealed the prevalence of unprotected neonates was 95.9% [21]. The re-emerge of pertussis in China was reported in several researches [22, 23]. According to our previous research, from November 2015 to May 2019, 5.0% (34/686) of cough neonates in neonatal ward of Beijing Children’s Hospital was diagnosed with pertussis (unpublished data). The hospitalized neonates in the present study were centralized management, the physicians should have awareness to prevent pertussis outbreak in neonatal ward. The missed pertussis cases would be, no doubt, an important source of ongoing transmission within the department. The physicians in clinical should include *B. pertussis* in routine screening and diagnostics in cough neonates.

We demonstrated that not only low protective level against DTP-associated antibodies, but also discordance of detectable rate of anti-Ptx between Shunyi and Qianjiang. The neonates from Qianjiang had lower detectable rate of anti-Dtx (57.5%) and anti-Ttx IgG (55.8%) than neonates from Shunyi (97.5% for anti-Dtx and 71.0% for anti-Ttx). As three components in one combined vaccine, the detectable rate of anti-Ptx should have the same pattern. However, the immunity after pertussis vaccination wanes with time, the amount of antibodies declines four years after receiving the last dose of the vaccine and after ten years will reach 0–20% [24]. Thus, theoretically, neonates from Qianjiang had lower or similar detectable rate of anti-Ptx. Much to our surprise, the detectable rate of anti-Ptx IgG in neonates from Qianjiang was higher significantly. The higher detectable rate of anti-Ptx IgG from Qianjiang suggested that pertussis is still likely circulating in this place. We could not eliminate the detectable rate of anti-Ptx IgG was induced by expose to pertussis of the mothers. In the current study, 2 neonates from Qianjiang have anti-PT IgG ≥100.0 IU/ml, which suggested that their mothers or they have a recent pertussis course. Considering the very low level of anti-Ptx IgG, the cut-off value of ≥100.0 IU/ml for recent pertussis infection might be relatively harsh. We could not eliminate that those mothers who had anti-Ptx IgG 40.0–100.0 IU/ml did have a real pertussis infection. Anti-Ptx IgA is an important marker of recent pertussis infection. Among all 7 neonates who had anti-PT IgG levels ≥40.0 IU/ml, none of them had detectable anti-PT IgA level. Therefore, we speculated that the mothers, not these 2 neonates (anti-PT IgG ≥100.0 IU/ml) had a recent pertussis course. Among 95 neonates from Qianjiang with detectable anti-Ptx IgG, 53 of them had undetectable anti-Dtx IgG and/or anti-Ttx IgG. This result also suggested that the higher detectable rate of anti-Ptx IgG maybe induced by expose to pertussis by mothers. Pertussis is still a public health problem in Qianjiang.

There were certain limitations in this study. One of the limitations of this study was the sample size and all subjects were collect in only two hospitals. An analysis of the distribution of DTP associated antibodies in neonates from different Chinese regions and provinces are required. Second, the information on the vaccination status of neonates’ mothers could not be obtained. It has been nearly 20 years since the mothers were vaccinated, and anti-Dtx and anti-Ttx were used as an indicator of DTP vaccination. Third, no maternal blood samples were collected in this study, this would be a very useful and complete comparison.

## Conclusions

The regional discrepancy of the protective antibody rates might be caused by different vaccine coverage and pertussis exposure, which suggested the importance of Tdap booster immunization for pregnant women or women at childbearing age, those living undeveloped areas in particular. More attention should be paid to immunization implement and control of infectious diseases in the remote region or lower income regions.

## Data Availability

The raw data will be provided upon request by Kaihu Yao (Correspondence author), Email: yaokaihu@bch.com.cn.

## Abbreviations

anti-Dtx: Anti-diphtheria
anti-Ptx: Anti-pertussis toxin
anti-Ttx: Anti-tetanus
CI: Confidence interval
DTaP: Diphtheria-tetanus-acellular pertussis
DTwP: Diphtheria-tetanus-whole cell pertussis
EPI: Expanded Program on Immunization
IgG: Immunoglobulin G
IU: International units
